# Knockdown of PKM2 enhances radiosensitivity of cervical cancer cells

**DOI:** 10.1186/s12935-019-0845-7

**Published:** 2019-05-14

**Authors:** Yanzhu Lin, Hui Zhai, Yi Ouyang, Zhiyuan Lu, Chengbiao Chu, Qianting He, Xinping Cao

**Affiliations:** 1 Department of Radiation Oncology, Sun Yat-sen University Cancer Center, State Key Laboratory of Oncology in South China, Collaborative Innovation Center for Cancer Medicine, Guangzhou, China; 2 Gynecology Department, Jinan Maternity and Child Care Hospital, Jinan, China; 3 Department of Oral and Maxillofacial Surgery, The First Affiliated Hospital, Sun Yat-Sen University, Guangzhou, China; 4 Department of Pathology, Sun Yat-sen University Cancer Center, State Key Laboratory of Oncology in South China, Collaborative Innovation Center for Cancer Medicine, Guangzhou, China

**Keywords:** Radiosensitivity, Apoptosis, Cell cycle, Double strand break repair

## Abstract

**Background:**

Pyruvate kinase isozyme type M2 (PKM2) catalyzes the final step in glycolysis and has been found to be up-regulated in multiple human malignancies. However, whether PKM2 regulates the radiosensitivity of human cervical cancer (CC) remains unknown.

**Methods:**

The expression of PKM2 in 94 patients with CC in the complete response (CR) and noncomplete response (nCR) groups, was evaluated by immunohistochemistry. The effect of PKM2 inhibition on radiosensitivity, the cell cycle, DNA damage, and apoptosis was evaluated by immunofluorescence analysis, colony formation assay, flow cytometry analysis and Western blotting.

**Results:**

PKM2 expression was more highly expressed in the nCR group than that in CR group and PKM2 expression was enhanced in CC cells after ionizing radiation (IR). In addition, knockdown of PKM2 combined with IR significantly reduced cell growth, promoted apoptosis, and enhanced radiosensitivity. Additionally, knockdown of PKM2 with IR resulted in increased phosphorylation of DNA repair checkpoint proteins (ATM) and phosphorylated-H2AX. Moreover, knockdown of PKM2 combined with IR significantly increased the expression of cleaved caspase 3 and caspase 9, whereas Bcl2 expression was suppressed. Furthermore, knockdown of PKM2 combined with IR markedly reduced the expression of several cancer stem cell biomarkers in vitro, including NANOG, OCT4, SOX2, and Bmi1.

**Conclusions:**

The results of our study suggests that PKM2 might be involved in mediating CC radiosensitivity and is identified as a potentially important target to enhance radiosensitivity in patients with CC.

**Electronic supplementary material:**

The online version of this article (10.1186/s12935-019-0845-7) contains supplementary material, which is available to authorized users.

## Background

Cervical cancer (CC) is the second most common gynecological cancer worldwide and seriously threatens women’s health [1]. Radiotherapy is one of the most effective modalities for the treatment of locoregionally advanced CC (LACC). Unfortunately, in some patients, cancer cells acquire radioresistance during radiotherapy, leading to local therapeutic failure, which is responsible for the high recurrence rate and poor survival among patients with CC [2]. Therefore, novel strategies intended to enhance the radiosensitivity of cancer cells and research on the underlying mechanisms of CC are essential.

Previous studies have shown that the high aerobic glycolysis rate of malignant tumors results in higher lactic acid production, which is closely related to metastasis and radioresistance of cancers [3, 4]. Additionally, other studies have found that inhibition of glycolysis increases radiosensitivity [5–7]. However, the mechanism through which inhibition of glycolysis increases radiosensitivity remains poorly understood. Pyruvate kinase isozyme type M2 (PKM2) is a rate-limiting enzyme in the final step of the glycolytic pathway, which catalyzes the conversion of phosphoenolpyruvate (PEP) and adenosine diphosphate (ADP) to pyruvate and is responsible for ATP production [8, 9]. Meng et al. [10] found that knockdown of PKM2 expression enhances the radiosensitivity of non-small-cell lung cancer cell lines through inhibition of AKT and PDK1 phosphorylation and increases the rate of ERK1/2 and GSK3β phosphorylation. Another study showed that overexpression of PKM2 is associated with radiation resistance in CC, but its molecular mechanism was not established [11]. We previously reported that PKM2 was up-regulated in CC and may serve as a molecular target [12]. However, the role of PKM2 in radiosensitivity of CC has not been elucidated.

It has been demonstrated that the presence of cancer stem cells (CSCs) in solid tumors is a major factor in radioresistance [13]. Treatment modalities targeting CSCs may substantially improve outcome in patients with cancer. Accordingly, recent studies have increasingly focused on the identification of CSC-specific markers, such as CD44, CD133, ABCG2 (CD338), and ALDH1 and stem cell transcription factors SOX2, OCT4, and NANOG. However, the molecular mechanism of radioresistance in CSCs remains unclear. A previous study showed that nuclear PKM2 expression positively correlates with stem-cell-like properties [14]. PKM2 has also recently been found to regulate gene transcription of OCT4 [15, 16]. Therefore, the role of PKM2 in cervical CSCs requires further exploration.

The main goal of this study was to examine the expression of PKM2 in association with radiation resistance in CC and to investigate the radiotherapy resistance mechanism at the molecular level.

## Materials and methods

### Sample collection

A total of 94 patients who had been treated with definitive radiotherapy for CC at Sun Yat-sen University Cancer Center (Guangzhou, China) from November 2016 to December 2017 were analyzed. The collection of human tissue samples was approved and supervised by the Ethics Committee of Sun Yat-sen University. All specimens had confirmed pathological diagnoses. Patients with distant metastasis were excluded. The patients were treated with definitive radiotherapy consisting of external beam radiotherapy (intensity-modulated radiotherapy) followed by high-dose-rate brachytherapy with or without platinum-based concurrent chemotherapy. Immunohistochemistry (IHC) was performed as previously described [12]. The proportion of PKM2 staining was graded as (1–25% = 1; 26–50% = 2; 51–75% = 3; > 76% = 4) and the staining intensity was graded as (negative = 0; weak = 1; moderate = 2; strong = 3). The final score for PKM2 expression was calculated by multiplication of these 2 scores. Slides were considered low or high, with scores of ≤ 7 or > 7, respectively.

### Response evaluation

The response to treatment was assessed according to the Response Evaluation Criteria in Solid Tumors after the completion of radiotherapy [17]. A complete response (CR) was defined as the disappearance of all target and nontarget lesions and no new lesions documented after two assessments at least 4 weeks apart. A partial response was defined as the detection of at least a 30% reduction in the sum of the longest dimensions of the target lesions in two assessments at least 4 weeks apart. Progressive disease was defined as a 20% increase in the sum of the longest dimensions of the target lesions or the development of new lesions. Stable disease implies that none of the previously mentioned statuses applied. At 3 months after treatment, all patients were divided into two groups: CR and noncomplete response (nCR) groups.

### Cell culture

The CC cell lines SiHa and HeLa were obtained from the American Type Culture Collection (Manassas, VA, United States) and were cultured in DMEM (Invitrogen, Carlsbad, CA, United States) or 1640 (Gibco; Gaithersburg, MD, United States) containing 10% fetal bovine serum, 100 µ/mL penicillin, and 100 µg/mL streptomycin at 37 °C in 5% humidified CO_2_. Stable knockdown PKM2 SiHa and HeLa cell lines were constructed as previously described [12].

### Cell viability assay

HeLa and SiHa cells were seeded at a density of 10^4^ cells/mL in 96-well plates and incubated at 37 °C overnight. Cells were treated with various doses of ionizing radiation (IR) (0, 2, 4, 6, and 8 Gy) using an RS2000 X-ray irradiator (Rad Source; Rad Source Technologies, Coral Springs, FL, United States) at a dose rate of 1.1 Gy/min. After treatment, the cells were incubated for an additional 48 h, and cell viability was determined using Cell Counting Kit-8 (Dojindo; Kumamoto, Japan) and by measuring absorbance at 450 nm using an ELx800™ plate reader (BioTek; Winooski, VT, USA) following the manufacturer’s instructions.

### Clonogenic cell survival assay for cell survival fraction analysis

Radioresponse was assessed using a colony formation assay. Different numbers of cells were seeded into six-well plates (150, 300, 600, 1000, and 2000 per well). The next day, cells were irradiated with 0, 2, 4, 6, and 8 Gy by an X-ray irradiator at room temperature. After IR, cells were incubated for 14 days to form colonies. Colonies were washed twice with PBS, fixed with methanol, and stained with crystal violet; colonies of more than 50 cells were then counted. Plating efficiency (PE) was calculated in triplicate as PE = (colony number/plating cell number) × 100%. The surviving fraction (SF) was estimated by calculating SF = colony number/(cells seeded × plating efficiency).

### Western blot

Total proteins were extracted as described previously [12]. Cells were lysed in radioimmunoprecipitation assay buffer (Beyotime; Haimen, China), and protein concentrations were measured through bicinchoninic protein assay kit (Pierce, Appleton, WI, USA). Equal amounts of protein were separated through SDS-PAGE and subsequently transferred to a PVDF membrane (Millipore; Burlington, MA, United States). After the membrane was blocked for 1 h using 5% skim milk, it was incubated with primary antibody overnight at 4 °C. Finally, the membrane was incubated with respective secondary antibodies (Santa Cruz, United States) for 1 h. Signals were detected using enhanced chemiluminescence reagents (Pierce; Waltham, MA, United States). Sources of antibodies and concentrations used were as follows: rabbit anti-PKM2 (1:1000, CST, USA), rabbit anti-phospho-ATM (Ser1981) (1:1000, CST, USA), rabbit anti-phospho-BRCA1 (Ser1524) (1:1000, CST, USA), rabbit anti-phospho-Chk1 (Ser345) (1:1000, CST, USA), rabbit anti-phospho-Chk2 (Thr68) (1:1000, CST, USA), rabbit anti-phospho-histone H2AX (Ser139) (1:1000, CST, USA), mouse anti-cycline B1 (1:1000, Santa Cruz, USA), mouse anti-phospho-p53 (Ser15) (1:1000, CST, USA), rabbit anti-cleaved caspase 3 (1:1000, CST, USA), rabbit anti-caspase 3 (1:1000, CST, USA), rabbit anti-cleaved caspase 9 (1:1000, CST, USA), mouse anti-caspase 9 (1:1000, CST, USA), rabbit anti-Bcl2 (1:1000, CST, USA), rabbit anti-Bax (1:1000, CST, USA), rabbit anti-NANOG (1:1000, Abcam, USA), rabbit anti-OCT4 (1:1000, CST, USA), rabbit anti-SOX2 (1:1000, CST, USA), rabbit anti-KLF4 (1:1000, CST, USA), mouse anti-ABCG2 (1:1000, CST, USA), mouse anti-Bmi1 (1:1000, Santa Cruz, USA), rabbit anti-GAPDH (1:1000, CST, USA). Glyceraldehyde 3-phosphate dehydrogenase (GAPDH) was used as the loading control. Quantity One Software (Bio-Rad) was used to analyze the intensity of blots.

### RNA extraction and quantitative RT-PCR

Total RNA was extracted from the HeLa and SiHa cells using TRIzol (Invitrogen; Milan, Italy) according to manufacturer’s protocol, and 500 ng was used to obtain cDNA through reverse transcription using PrimeScript RTMasterMix (Promega; Madison, WI, United States). Quantitative RT-PCR was conducted using an SYBR Green PCR master mix (Roche; Basel, Switzerland) on the CFX96 Real-Time PCR Detection System (Bio-Rad; Hercules, CA, United States). PCR amplification was performed with the specific primer sets as described previously [12]. Relative expression was normalized to the expression of β-actin. The 2^−ΔΔCt^ method was used for relative quantification of gene expression. The primers used in the studies were: PKM2 sense, 5′-ATGTCGAAGCCCCATAGTGAA-3′, PKM2 antisense, 5′-TGGGTGGTGAATCAATGTCCA-3′, β-actin sense, 5′-CTCCATCCTGGCCTCGCTGT-3′, β-actin antisense, 5′-GCTGTCACCTTCACCGTTCC-3′.

### Cell cycle analysis

HeLa and SiHa cells that were irradiated with 2 Gy were collected at 24 h after IR and assessed for cell cycle distribution through flow cytometry. Briefly, 10^6^ cells were harvested with trypsin and fixed in 70% ethanol for 24 h. The cells were then washed and suspended in 500 μL of PBS buffer containing 25 mg/mL RNAse and 50 μg/mL propidium iodide and incubated for 15 min in the dark. The cells were subsequently detected through flow cytometry (Beckman Coulter, Inc.; Brea, CA, US), and the data were analyzed by Modifit (Beckman Coulter).

### Apoptotic assays

Apoptotic cell death was assessed through flow cytometry using the Annexin V-APC/7-AAD Apoptosis Detection Kit (BestBio; Shanghai, China) according to the manufacturer’s instructions. Briefly, the indicated cells were exposed to 0 or 2 Gy of IR. After treatment, cells were incubated at 37 °C for 48 h. Then, 10^5^ cells were resuspended in 500 μL of 1× binding buffer and stained with 10 μL of Annexin V-APC and 5 μL of 7-AAD. Apoptosis levels were detected through flow cytometry (Beckman Coulter).

### Immunofluorescence

Immunofluorescence detection of phospho-histone-H2AX (γ-H2AX) foci was performed to monitor formation of double-strand DNA breaks (DSBs). At specified time points (1, 12, and 24 h) after treatment with 2 Gy, cells were fixed with 4% paraformaldehyde for 15 min, permeabilized with 0.2% Triton X-100 for 5 min, blocked with 5% bovine serum albumin in PBS, and incubated overnight at 4 °C with γ-H2AX (Ser139, 1:1000, CST) antibody. For visualization, cells were incubated with Alexa Fluor 488 conjugated goat antirabbit secondary antibody (Abcam; Cambridge, UK; 1:1000) for 1 h. DAPI (Sigma-Aldrich; St. Louis, MO, United States; 1:3000) was used as a nuclear counterstain. Foci of γ-H2AX were counted from at least five random fields under a Leica Confocal Laser Scanning Microscope (Leica Microsystems; Wetzlar, Germany).

### Statistical analysis

Each experiment was performed in triplicate. Data are expressed as mean ± SD (standard deviation). The statistical analysis was performed using SPSS 20.0 (SPSS, Inc.; Chicago, IL, United States). The significance of differences between two groups was determined by the t-test and One-way analysis of variance was used for multiple comparisons. *P* < 0.05 was defined as statistically significant.

## Results

### PKM2 is involved in tumor radiosensitivity

**Fig. 1:**
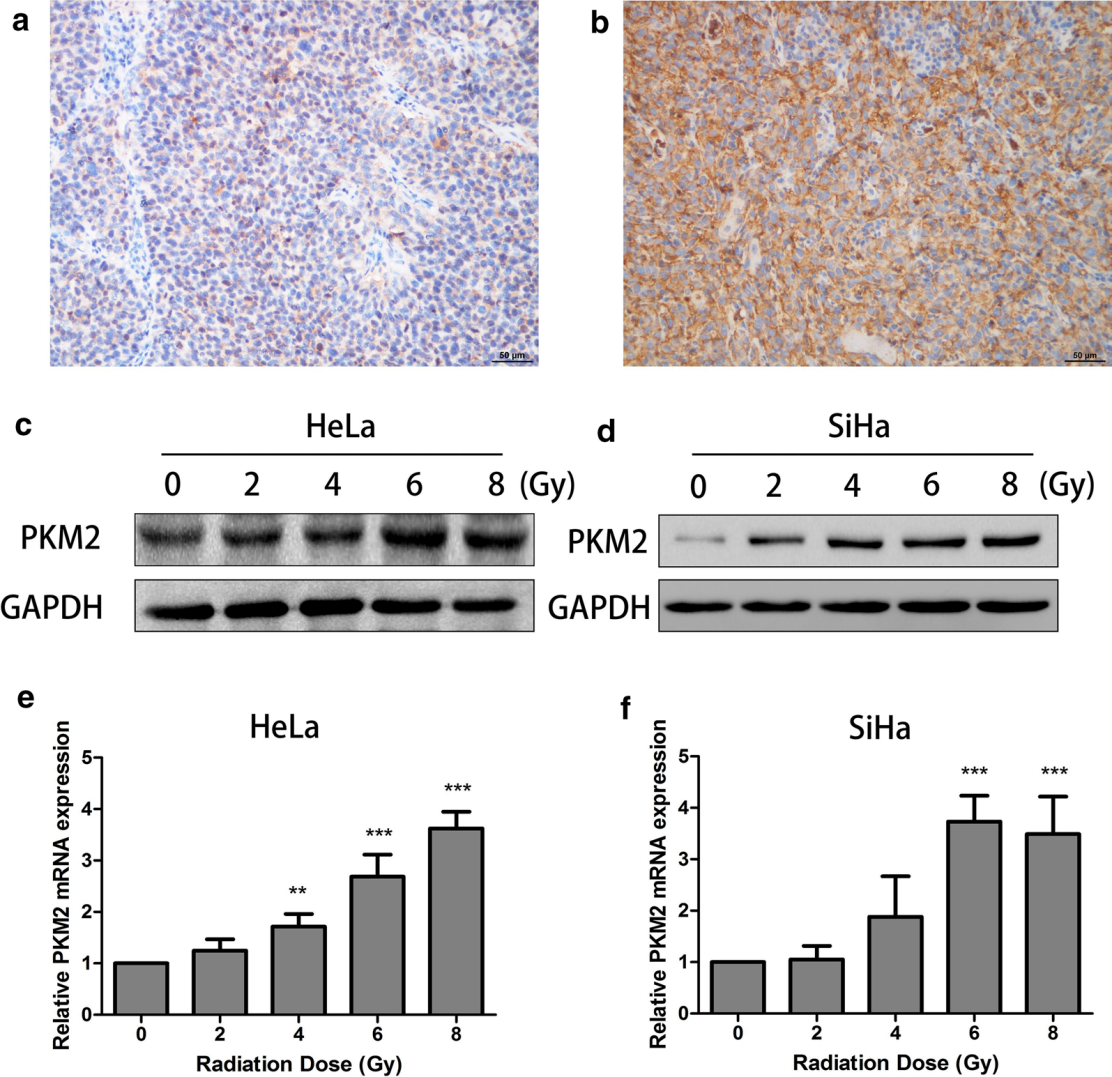
PKM2 expression was associated with radiation resistance. Representative examples of PKM2 staining of cervical cancers in the radiation-resistant group and radiation-sensitive group. **a** Weak positive staining of PKM2 in the radiation-sensitive group. **b** Strong positive staining of PKM2 in the radiation-resistant group. **c**, **d** HeLa and SiHa cells were cultured for 24 h after 0, 2, 4, 6, and 8 Gy of X-ray irradiation. Total protein were extracted and analyzed through Western blot. **e**, **f** HeLa and SiHa cells were cultured for 24 h after 0, 2, 4, 6, and 8 Gy of X-ray irradiation. Total RNA were extracted and analyzed through quantitative RT-PCR. Data represent three independent experiments. **P < 0.01, ***P < 0.001 compared with the 0 Gy group

PKM2 expression in CC tissues was detected by IHC staining. In total, 36 (38%) patients had CR, and 58 patients (62%) had nCR (Table 1). The results revealed that the PKM2 expression in the radiation-resistant group was statistically significantly higher than that in the radiation-sensitive group (Fig. 1a, b, Table 1, *P* = 0.002). However, there were no statistically significant differences between the radiation response and the clinicopathological features (Additional file 1: Table S1). These results suggested that PKM2 is clinically associated with resistance to radiation.

**Table 1:** Relationship between PKM2 expression and response to radiotherapy

Group	Patients (n = 94)	PKM2 expression		*P*-value
		Low-expression (n = 46)	High-expression (n = 48)	
Radiation-sensitive	36	25	11	0.002
Radiation-resistant	58	21	37

To test this hypothesis, we first treated HeLa and SiHa cells with increasing doses of IR. Then, we showed, by Western blot and quantitative RT-PCR analyses, that the expressions of PKM2 increased after exposure to different radiation doses in cell lines (Fig. 1c, d), suggesting that PKM2 might potentially play a role in modulating radiosensitivity of CC cells.

### Knockdown of PKM2 enhanced cell radiosensitivity

**Fig. 2:**
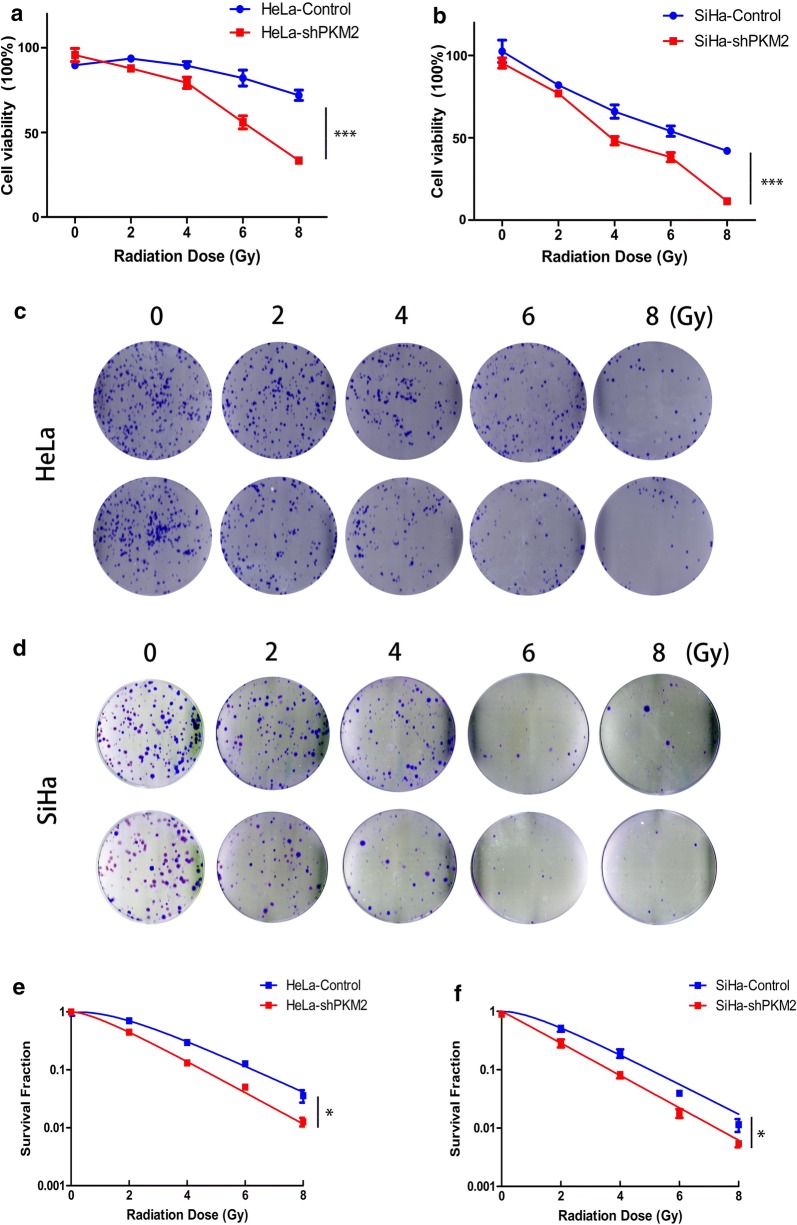
Knockdown of PKM2 expression enhanced the radiosensitivity of HeLa and SiHa cells in vitro. **a**, **b** Cells were irradiated at different doses of irradiation, and the CCK-8 assay was used to test the viability of these cells. **c**, **d** Radiation sensitivity was tested using colony formation assays. Knockdown of PKM2 decreased clonogenic formation in HeLa and SiHa cells compared with control. **e**, **f** Survival curves were obtained using a multitarget click mathematical model. Data represent three independent experiments. *P < 0.05, ***P < 0.001

We also examined the role of PKM2 in radioresponsiveness in HeLa and SiHa cells. Stable low PKM2 expression in HeLa and SiHa cell lines was established using a lentiviral expression system as previously reported [12]. The results of the cell viability assay showed that the viability of PKM2 knockdown cells was significantly lower than that of control cells after exposure to various doses of IR (Fig. 2a, b). To further validate the effect of PKM2 silencing on radiosensitivity, colony formation ability was assayed after IR exposure. The number of colonies formed by PKM2-shRNA cells was significantly decreased compared with that of control cells (Fig. 2c, d). These results suggested that knockdown of PKM2 made cells more sensitive to IR compared with the control cells. Similar results were obtained with SiHa cells. SF curves also revealed that the clonogenicity of PKM2-shRNA group cells was dramatically reduced in an ionizing radiation-dose-dependent manner (Fig. 2e, f). These results suggested that PKM2 inhibition can enhance radiosensitivity in CC cells.

### Knockdown of PKM2 increased DNA damage

**Fig. 3:**
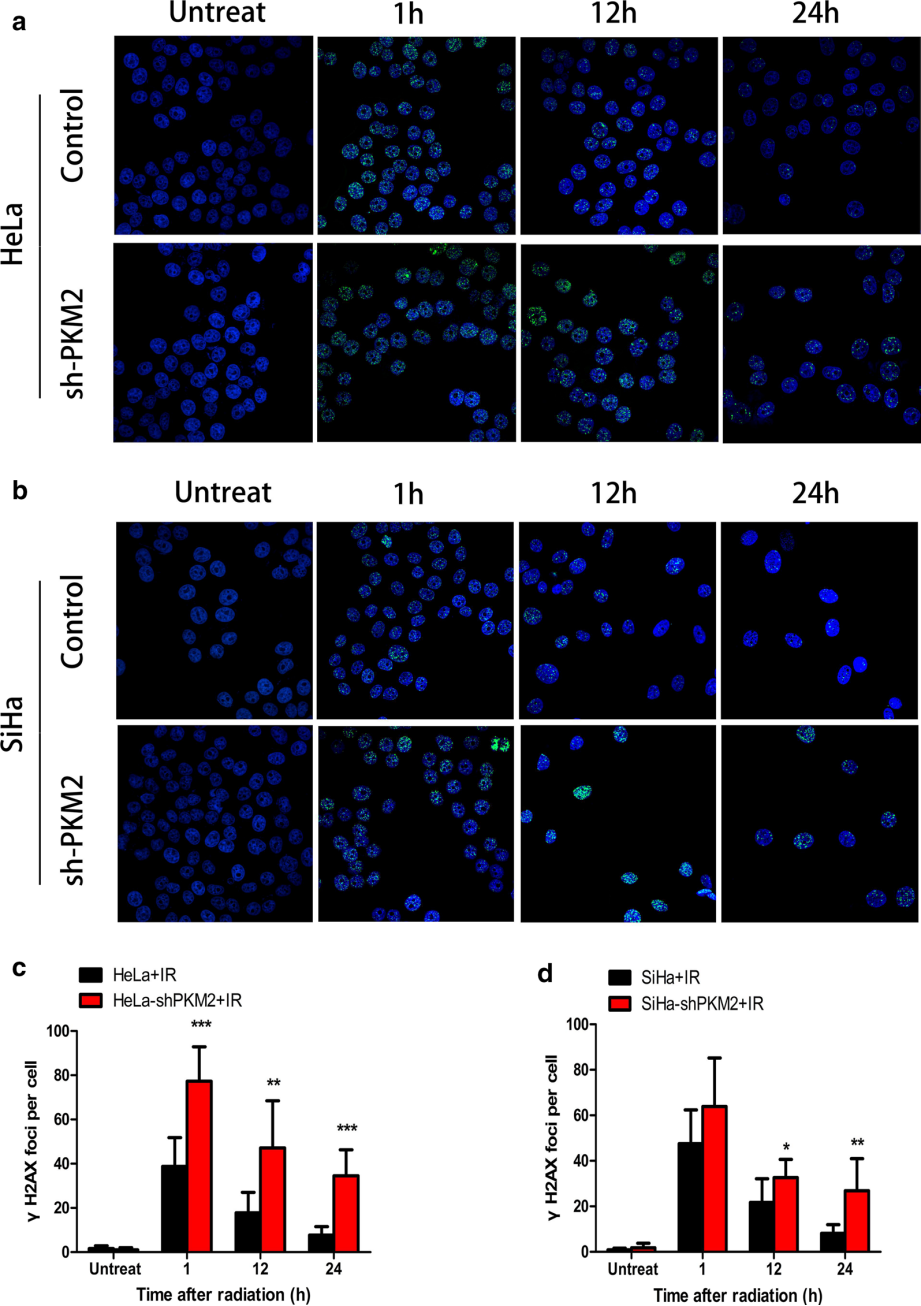
PKM2 influenced DNA damage repair. **a**, **b** Different group of cells was subjected to IR. 1, 12, and 24 h after IR, cells were fixed and immunostained for γ-H2AX foci. The numbers of γ-H2AX foci at all time points were measured in both HeLa and SiHa cells. Untreated cells were used as the negative control. Representative images are presented here (400×). **c**, **d** Knockdown of PKM2 significantly increased the numbers of γ-H2AX foci after different doses of irradiation in both HeLa and SiHa cells. Data represent three independent experiments. *P < 0.05; **P < 0.01; ***P < 0.001

The phosphorylated form of H2AX, γ-H2AX, has been identified as a marker of the early response to DNA damage [18]. To determine whether knockdown of PKM2 regulates radiation-induced DNA DSBs, the number of γ-H2AX foci at different times after IR (2 Gy) was counted. As shown in Fig. 3, the number of γ-H2AX foci in the control group increased at 1 h after IR, and decreased rapidly at 24 h after IR. In contrast to the control group, number of γ-H2AX foci decreased more slowly in the PKM2 silencing group (Fig. 3a, b). The data revealed that knockdown of PKM2 increased DNA damage in response to ionizing radiation treatment.

### Knockdown of PKM2 promoted radiosensitivity by arresting cells in the cell cycle G2/M phase and inducing apoptosis

**Fig. 4:**
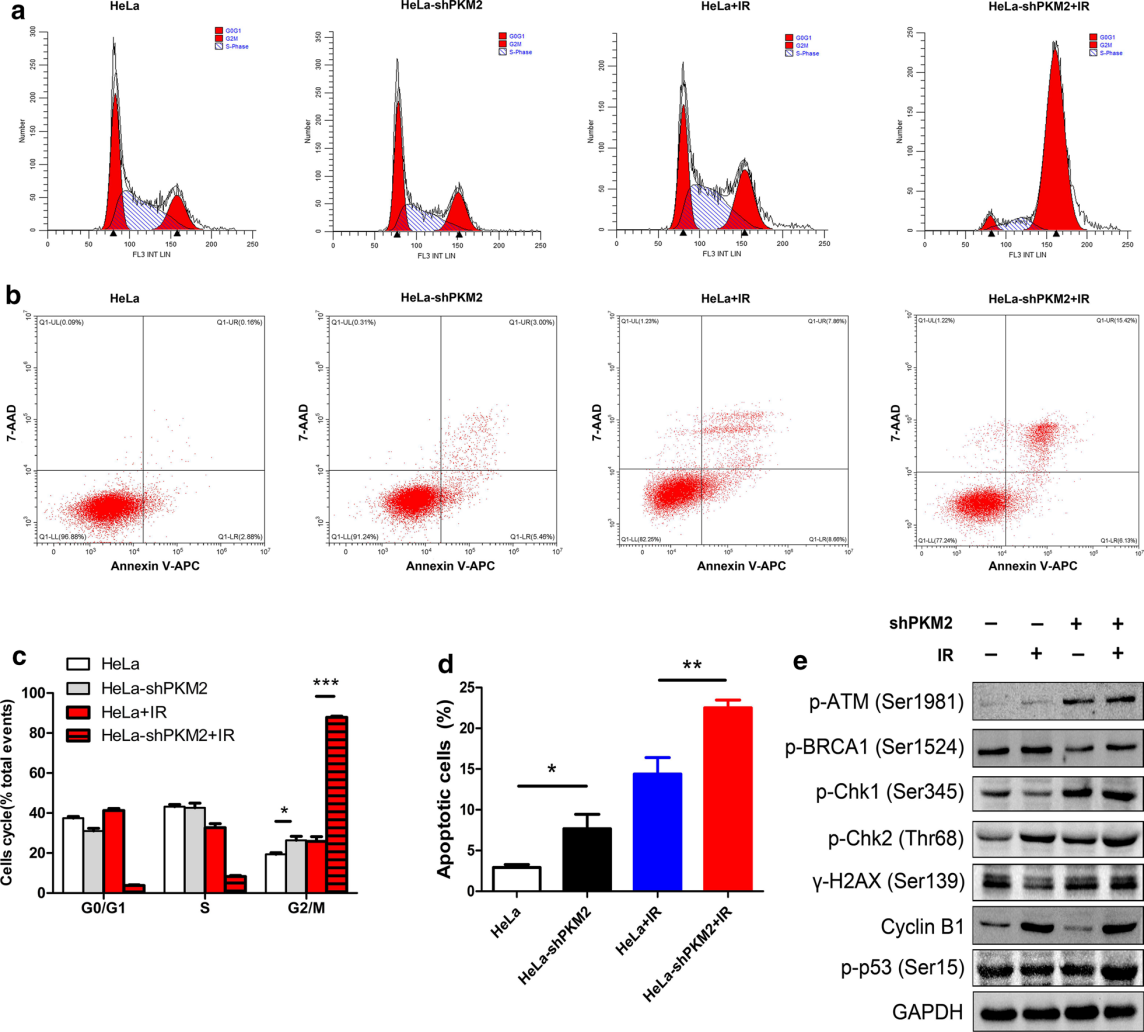
Knockdown of PKM2 induced G2/M-phase arrest and apoptosis. **a** Cell cycle distribution was measured through flow cytometry. Graphic representation of flow cytometry data showing percentage of cells in G1, S, and G2/M phase. **b** After irradiation (IR), cells were incubated for 48 h and measured through Annexin V-APC/7-AAD staining and flow cytometry. Knockdown of PKM2 significantly promoted cell apoptosis after radiation treatment. **c** Graphic representation of flow cytometry data showing percentage of cells in G1, S, and G2/M phases. *P < 0.05, ***P < 0.001. **d** Statistical images of cell apoptosis (*P < 0.05, **P < 0.01). **e** The proteins p-ATM, p-BRCA1, p-Chk1, p-Chk2, Cyclin B1, p-P53, and γ-H2AX were detected through Western blotting. GAPDH was used as an internal reference. Data represent three independent experiments. *P* phosphate

The effect of PKM2 inhibition on cell cycle distribution was investigated. The cell cycle distribution of HeLa and SiHa cells revealed that PKM2 knockdown significantly increased the proportion of cells in the G2/M phase (Fig. 4a and Additional file 2: Figure S1A and C). We also analyzed the role of PKM2 in the cell cycle in response to radiation. The proportion of the shPKM2 + radiation group cells in the G2/M phase was significantly higher compared with that of the control group in HeLa and SiHa cells (Fig. 4c and Additional file 2: Figure S1A and C). The data suggest that knockdown of PKM2 induced an accumulation of cells, indicative of a G2/M arrest after IR.

We analyzed the effect of PKM2 knockdown on IR-induced cell apoptosis. The apoptosis levels were significantly increased in the HeLa PKM2-shRNA groups (Fig. 4b). In addition, a significantly increased apoptosis rate was observed in HeLa PKM2-shRNA cells after IR compared with the control group. This indicates that PKM2 inhibition enhances radiation-induced apoptosis. Additionally, knockdown of PKM2 in SiHa cells significantly enhanced radiation-induced cell apoptosis (Additional file 2: Figure S1B and D). According to these findings, knockdown of PKM2 promoted radiosensitivity by enhancing IR-induced apoptosis of CC cells.

We additionally evaluated the levels of cell cycle regulatory protein to determine the effect of PKM2 on radiation-induced DNA damage and repair. As shown in Fig. 4e, in HeLa cells, the levels of the p-ATM, p-Chk1, and p-Chk2 proteins, which are key checkpoint proteins, are higher in sh-PKM2 cells than in control cells, whereas cyclin B1 levels are decreased. Also, knockdown of PKM2 in HeLa cells led to upregulation of phospho-ATM, Chk1, p53, and γ-H2AX, after IR treatment. These findings indicated that the DNA damage checkpoint response was activated.

### Knockdown of PKM2 reduced CSC transcription factors after IR

**Fig. 5:**
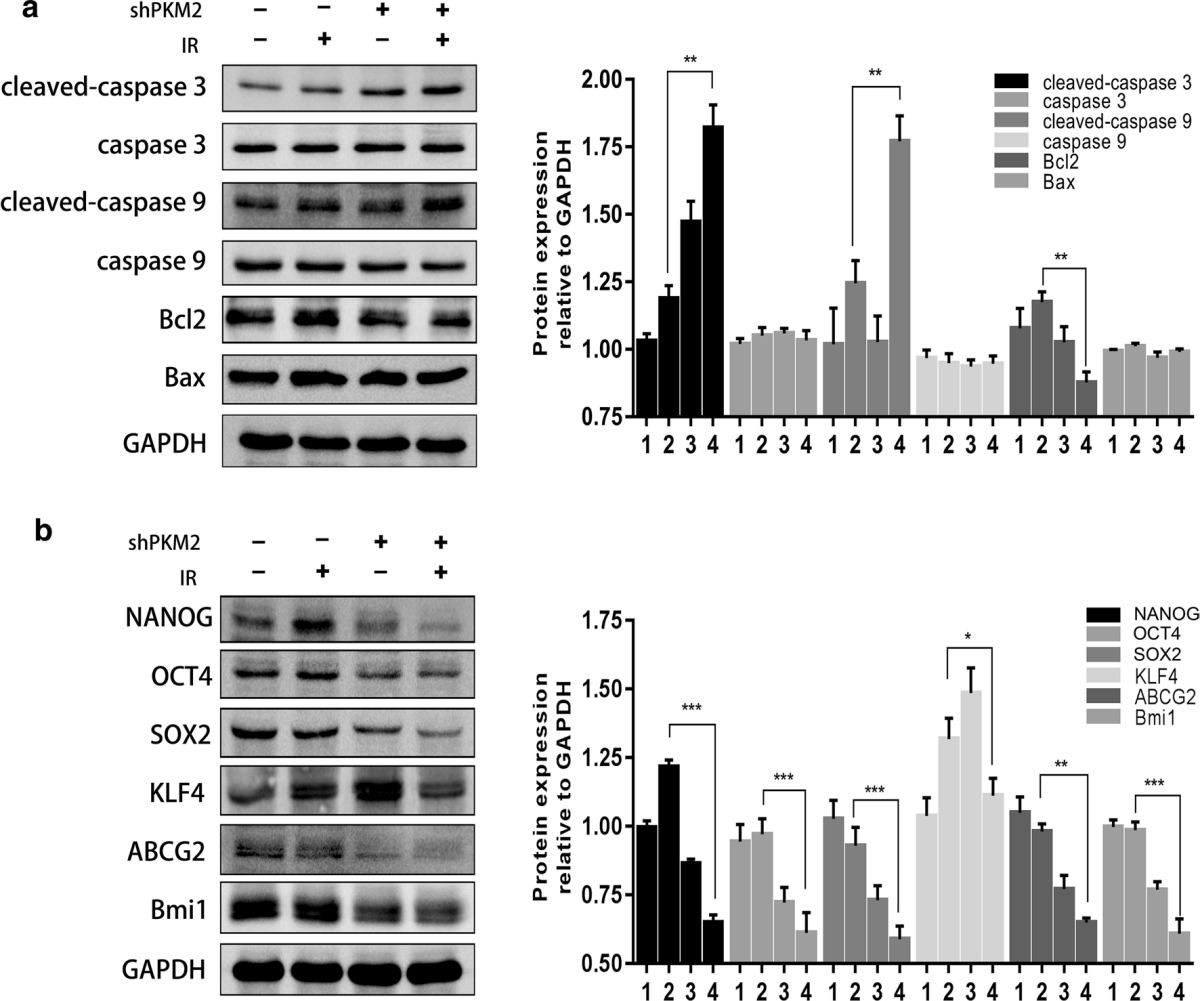
Western blot for the detection of stemness-associated markers. **a** Cells were pretreated with or without 2 Gy of IR and were analyzed for the expression of cleaved caspase 3, cleaved caspase 9, caspase 3, caspase 9, Bax, and Bcl2 protein levels through Western blotting. **b** Knockdown of PKM2 combined with IR downregulated NANOG/OCT4/SOX2 in protein expression. GAPDH protein was used as an internal standard. Data represent three independent experiments. *P < 0.05; **P < 0.01; ***P < 0.001. (shPKM2: shRNA against PKM2; IR: irradiation. 1 = control cells, 2 = control cells + IR, 3 = shPKM2 cells, 4 = shPKM2 cells + IR)

To elucidate the molecular mechanism of PKM2-mediated radiosensitivity of CC cells, the levels of several apoptosis-related proteins were measured in cells following radiation treatment. As shown in Fig. 5a, PKM2 inhibition increased the expression of cleaved caspase 3 and cleaved caspase 9 and reduced Bcl2 expression in irradiated HeLa cells (Fig. 5a), which contribute to apoptosis after IR.

Since CSCs and radioresistance are related, we also evaluated the effect of PKM2 silencing on the expression of stemness-associated transcription factors (KLF4, SOX2, OCT4, ABCG2, and NANOG). The results showed a drastic decrease in the levels of SOX2, OCT4, ABCG2, Bmi1, and NANOG in shPKM2 HeLa cells compared with those in the control HeLa cells. Additionally, the expression levels of SOX2, OCT4, ABCG2, Bmi1, and NANOG in shPKM2 HeLa cells were also significantly reduced compared with the expression levels in the controls after irradiation (Fig. 5b). These results indicated that depletion of PKM2 leads to reduced expression of CSC biomarkers.

## Discussion

Radiation therapy has become a vital tool for LACC treatment, but most patients develop local recurrence within 5 years of radiotherapy due to the acquisition of radioresistance [19]. To improve the efficacy of radiotherapy, research that focuses on tumor markers of radiosensitivity has become a major area of development in the field. We evaluated whether PKM2 is a radiosensitivity marker that modulates the response to IR. Remarkably, we found that knockdown of PKM2 inhibited cell growth, increased DNA damage, led to G2/M cell cycle arrest, accompanied by activation of p53, reduced expression of CSC markers, and thereby enhanced radiosensitivity.

The Warburg effect is a common phenomenon in most cancer cells that supports tumor cell growth, even in the presence of ample O_2_ [20]. Research studies have increasingly found that the Warburg effect is implicated in radiation as well as chemotherapy resistance [21, 22]. A previous study [23] revealed that cancer cells recovering from damage undergo mitochondrial restructuring and show increased aerobic glycolysis. Increasing evidence suggests that PKM2 plays a critical role in aerobic glycolysis and that knockdown of PKM2 in cancer cells reduces glucose uptake, increases oxygen consumption, and reduces lactate production to suppress the Warburg effect [24, 25]. The role of PKM2 in various cancers has been previously investigated [26], but its function in CC has not been fully elucidated. We previously reported that PKM2 is upregulated and suggested that it functions as a tumor promoter in human CC [12]. More importantly, it has been found that downregulation of PKM2 effectively enhances radiosensitivity in human non-small-cell lung carcinoma [10, 27] and glioblastoma multiforme cell lines (U87, T98G, and U251) [28]. As anticipated, our results showed that high PKM2 expression was related to clinical radioresistance in patients with CC. Additionally, exposure of cells to IR increased PKM2 expression suggested that elevated PKM2 expression might contribute to radiation resistance. Moreover, PKM2 inhibition enhanced radiosensitivity of CC in vitro through inhibition the of survival rate and induction of G2/M arrest followed by radiation-induced apoptosis.

The mechanism of radiation resistance is complex, and the underlying mechanism of the direct association between PKM2 expression and radiation resistance is not fully understood. A crucial effect of ionizing radiation is the alteration of cell cycle progression, resulting in cell cycle arrest in either G1, S, or the G2/M phases, and the fact that cells in different phases exhibit different radiosensitivity [29]. Cells in the M and G2 phases are the most radiosensitive, those in the G1 phase are less sensitive, and S-phase cells are the most resistant to IR [30]. IR-induced DNA damage results in the activation of the DNA repair mechanism, which is one of the most influential biological processes contributing to radioresistance. H2AX is phosphorylated at the sites of DNA DSBs induced by ionizing radiation and is required for recruitment of repair factors into the nuclear foci after DNA damage [31]. Accordingly, the function of H2AX is believed to be associated primarily with DNA damage. ATM is a key protein kinase which plays a crucial role in the cellular response to IR-induced DNA damage [32]. Checkpoint kinases 1 and 2 (Chk1 and Chk2) have emerged as critical mediators in cell cycle checkpoint control and facilitate cell cycle arrest. Previous studies have shown that activation of the checkpoint kinases results in accumulation of p53, which subsequently modulates the transcription of many proapoptotic genes, thus regulating IR-induced apoptosis [33–35]. In this study, we found that knockdown of PKM2 increases the phosphorylation of ATM, and Chk1 and induces G2/M cell cycle arrest in CC cells. Sizemore et al. [28] also found that ATM phosphorylates PKM2 at T328, and directly regulates DSB repair to cause radiation resistance. However, another study demonstrated that PKM2 interferes with the kinase activity of ATM toward P53 through a potential direct interaction [36]. Therefore, more research is required to clarify the how PKM2 regulates ATM and Chk1 activation. We also found that PKM2 inhibition induced a significant G2/M cell cycle arrest and activated p-p53 expression in irradiated CC cells, indicating that DNA damage was not repaired and culminates in apoptotic cell death. In addition, PKM2 induced caspase 3-independent apoptosis of CC cells. The Bcl‐2 family of proteins, including proapoptotic and antiapoptotic members, is the most influential mediator of cell apoptosis. Our results also indicated that Bcl‐2 expression decreases after IR. Thus, PKM2 knockdown promotes IR‐induced apoptosis by regulating Bcl‐2 and caspase 3 expression, leading to enhanced radiosensitivity.

The existence of CSCs has been implicated in cancer recurrence, which results in cancer treatment failures. Certain proteins, such as OCT4, SOX2, NANOG, and KLF4, are crucial transcription factors for the maintenance of stemness. It has been reported that radioresistance of CC is associated with CSCs [37]. A previous study found that PKM2 is vital for maintaining stem-cell-like properties [38]. Moreover, EGFR has been found to directly interact with PKM2 to regulate the transcription of stemness-related genes and promote the stem-like phenotype, thereby promoting invasion and metastasis [14]. Lee et al. [39] reported that PKM2 modulates the OCT4-dependent transactivation. Among the CSC markers, depletion of NANOG alone is sufficient to reduce the proportion of CSCs. OCT4 and SOX2 are important transcriptional factors, and their expression has been reported to correlate with tumorigenesis, chemoresistance, and maintenance of the stem-cell-like phenotype in cancer cells [40–42], including CC cells [43]. High expression of SOX2 and OCT4 indicates radiation resistance in cervical squamous-cell carcinoma [44]. We also found that knockdown of PKM2 in CC cells reduced the expression of the CSC-related transcription factors NANOG, SOX2, OCT4, Bmi1, and KLF4.

## Conclusions

In summary, knockdown of PKM2 enhanced radiation sensitivity in CC cells by promoting cell apoptosis, inducing cell cycle arrest at the G2/M phase, thereby increasing radiation-induced DSBs. Such a mechanism may be used to reduce expression of stemness-related genes in CC cells. The results of this study indicate that targeting PKM2 may be a novel potential therapeutic option to increase the radiosensitivity of CC.

## Additional files

**Additional file 1: Table S1.** Patient characteristics

**Additional file 2: Figure S1.** Knockdown of PKM2 induces a G2/M cell cycle arrest and apoptosis in SiHa cells. (A) Cell cycle distribution was measured through flow cytometry. Graphic representation of flow cytometry data showing percentage of cells in G1, S, and G2/M. (B) Knockdown of PKM2 significantly promoted cell apoptosis after radiation treatment in SiHa cells. (C) Graphic representation of flow cytometry data showing percentage of cells in G1, S, and G2/M phases. *P < 0.05, **P < 0.01. (D) Statistical image of cell apoptosis, *P < 0.05; **P < 0.01; ***P < 0.001.

## Data Availability

All data generated or analysed during this study are included in this published article and its Additional files.

## Abbreviations

PKM2: pyruvate kinase isozyme type M2
CC: cervical cancer
LACC: locoregionally advanced CC
PEP: phosphoenolpyruvate
CSCs: cancer stem cells
CR: complete response
IR: ionizing radiation
IHC: immunohistochemistry
nCR: noncomplete response
PE: plating efficiency
SF: surviving fraction
SD: standard deviation
DSBs: double-strand DNA breaks
Chk1: checkpoint kinases 1
Chk2: checkpoint kinases 2
