# Supplementary material for: Knockdown of PKM2 enhances radiosensitivity of cervical cancer cells
Source: Cancer Cell Int. 2019 May 14;19:129. doi: 10.1186/s12935-019-0845-7 (PMC6518815; doi:10.1186/s12935-019-0845-7)
Supplement: Supplementary file 1 — Additional file 1: Table S1. Patient characteristics [file 12935_2019_845_MOESM1_ESM.doc]

Table. S1 Patient characteristics

| Characteristics | Patients  （n=94） | Radiation-resistant  (n = 58) | Radiation-sensitive  (n = 36) | *p*-Value |
| --- | --- | --- | --- | --- |
| Age (y) |  |  |  |  |
| ＜55 | 42 | 26 | 16 | 0.971 |
| ≥55 | 52 | 32 | 20 |  |
| FIGO stage |  |  |  |  |
| I+IIB | 55 | 31 | 24 | 0.206 |
| IIIA+IVA | 39 | 27 | 12 |  |
| Histological grade |  |  |  |  |
| Low+middle | 40 | 28 | 12 | 0.154 |
| high | 54 | 30 | 24 |  |
| Tumor size(cm) |  |  |  |  |
| ＜4.0 | 37 | 22 | 15 | 0.719 |
| ≥4.0 | 57 | 36 | 21 |  |
| Pelvic lymph node |  |  |  |  |
| (-) | 51 | 31 | 20 | 0.842 |
| (+) | 43 | 27 | 16 |  |
