# Supplementary material for: Knockdown of PKM2 enhances radiosensitivity of cervical cancer cells
Source: Cancer Cell Int. 2019 May 14;19:129. doi: 10.1186/s12935-019-0845-7 (PMC6518815; doi:10.1186/s12935-019-0845-7)
Supplement: Supplementary file 2 — Additional file 2: Figure S1. Knockdown of PKM2 induces a G2/M cell cycle arrest and apoptosis in SiHa cells. (A) Cell cycle distribution was measured through flow cytometry. Graphic representation of flow cytometry data showing percentage of cells in G1, S, and G2/M. (B) Knockdown of PKM2 significantly promoted cell apoptosis after radiation treatment in SiHa cells. (C) Graphic representation of flow cytometry data showing percentage of cells in G1, S, and G2/M phases. *P < 0.05, **P < 0.01. (D) Statistical image of cell apoptosis, *P < 0.05; **P < 0.01; ***P < 0.001. [file 12935_2019_845_MOESM2_ESM.pdf]

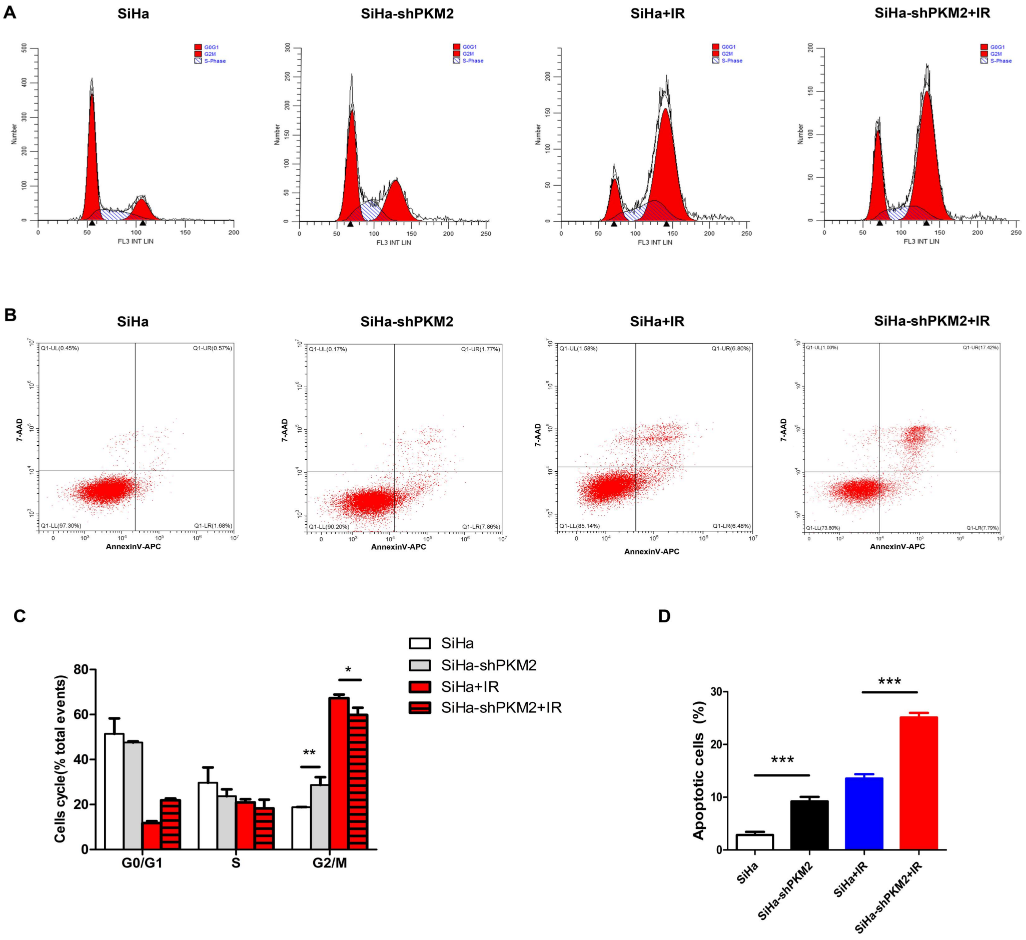

Figure S1: Knockdown of PKM2 induces a G2/M cell cycle arrest and apoptosis in SiHa cells. (A) Cell cycle distribution was measured through flow cytometry. Graphic representation of flow cytometry data showing percentage of cells in G1, S, and G2/M phases. (B) Knockdown of PKM2 significantly promoted cell apoptosis after radiation treatment in SiHa cells. (C) Graphic representation of flow cytometry data showing percentage of cells in G1, S, and G2/M phases. *P < 0.05, **P < 0.01. (D) Statistical image of cell apoptosis , ***P < 0.01.
